# Lime Inhalation in Croup

**Published:** 1872-05-01

**Authors:** J. P. Mathews

**Affiliations:** Carlinville, Ill.


### LIME INHALATION IN CROUP.

BY J. P. MATHEWS, M.D., CARLINVILLE, ILL.

The inhalation of lime in croup, as suggested to me by my friend, Dr. E. E. Webster, of Carondelet, Mo., has succeeded admirably in several cases. I use it by breaking lumps of fresh lime into pieces as large as eggs, and dropping them one by one into a bucket half-full of hot water, which causes ebullition, and from which the child inhales the steam. It should sit upon its nurse's or parent's knee, with a blanket thrown over both, and inhale the steam until it perspires freely, the pulse becomes weak, and the child presents some symptoms of exhaustion. It should then be wrapped in a dry blanket, and laid on a bed in a room pregnant with the same vapor. This should be repeated every three or four hours, until breathing becomes natural. This may be done independently of any other favorite treatment a physician trying it may choose. I have succeeded with it in several cases of true as well as diphtheritic croup, in which my experience has taught me I should have failed with any other mode. I therefore ask my professional brethren to give it a trial and report their success. I believe it will prove invaluable to many, as it has been to me, in treating this dreaded disease.

Indications for the Employment of Catheter in Old People.—M. Guyon, in a clinic at Hospital Necker, remarked that retention of urine is very common in old men, depending generally on affections of the bladder, or of the neck of the bladder, or of the prostate. Many cases of supposed vesical paralysis are, in reality, due to the prostatic disease. The symptoms of retention of urine in old people are eminently variable. Sometimes they micturate frequently, and experience pain and a burning heat, lasting for a long time. Again, where the bladder is but little contractile, the retention is only made out by percussive palpation and catheterism. When in this latter condition, they experience so little trouble that they are loth to submit to an exploratory operation. If the symptoms are well marked, then there is no room for hesitation; and M. Guyon even goes so far as to say that the catheter should be passed in every old man who evacuates the contents of his bladder imperfectly. It is not necessary that it should enter the organ on the first occasion, since if only introduced as far as the neck, it habituates the tissues to the contact of the instrument. Stoppage of the flow of water is always a serious symptom in old people, and the best advice to give them is to be sounded, either with simple sound or a catheter, and that frequently. When the bladder is greatly distended, it is imprudent to evacuate it completely. Commonly, the catheter should be passed only when there is an intense desire to urinate.—*Phil. Med. and Surg. Reporter.*

Herniopuncture.—In a paper read before the Louisville Medico-Chirurgical Society, Dr. Morton describes a new and easy mode for treating strangulated hernia. He evacuates the contents of the hernial tumor with a canula needle. Those used with hypodermic syringes will do very well, but one having a slight and uniform taper from heel to point is better. The tumor ought to be firmly grasped and steadied with one hand, and the instrument plunged suddenly and boldly in with the other, at least to the extent of half the diameter of the hernia, in the direction the needle is thrust, which should be toward the stricture backward and upward, This latter point is important, as it often happens that a very large quantity of bloody serum is effused into the space between the limits of the sac and the contained loop of intestine, making it necessary to plunge the needle deeply in order to reach the latter at all. The instrument must be allowed to remain until the gas ceases to flow out, when the operator should gently press the tumor, and at the same time slowly withdraw the instrument. By keeping up the pressure, if no adhesions exist, the loop of intestine will return to the cavity.—*Richmond and Louisville Med. Jour.*

A New Plan for Dressing Wounds.—The latest novelty in the mode of dressing wounds following amputation or other causes, is reported to *The Lancet* by its Paris correspondent. It originated with M. Alphonse Guerin, and consists in introducing cotton-wool into the stump or wound immediately. The amputated limb is then wrapped round with dry cotton-wool, a bandage being then applied, and tightened a little on subsequent days, if necessary, to maintain mild compression. The dressing, however, remains undisturbed until the twentieth or the twenty-fifth day, when on removing the packet of wadding, a cupful of pus is found in the folds of the cotton, and the wound is discovered to be quite healed. Notwithstanding the high mortality which existed during the German siege, M. Guerin obtained six successful results out of nine amputations of the thigh, treated with this method, and all of his cases of amputation of the leg did well.—*Medical Record.*

Treatment of Hydrocele.—Mr. Monod treats hydrocele by first drawing off a drachm or more of the liquid, and then injects alcohol either pure or dilute. The operation is repeated if necessary. He has cured in this way three cases of hydrocele, and a serous cyst of the thyroid. He suggests the same method for other serous accumulations.—*Ibid.*

Strangulated Hernia of 112 Hours' Duration.—Dr. Geo. Hill operated for the relief of a strangulated femoral hernia of the above duration. The patient was 78 years old, yet at the end of three weeks he was about his room, and finally recovered completely.—*Phil. Med. and Surg. Reporter.*

Carbolic Acid in Gunpowder Burns.—Dr. Walcott has used carbolic acid in gunpowder burns, and finds that in addition to its antiseptic and anæsthetic properties, it has the power of suspending the carbon in solution and withdrawing it from the skin, so that there is no discoloration remaining.—*Edinburgh Med. Jour.*

